# Waste to value: Global perspective on the impact of entomocomposting on environmental health, greenhouse gas mitigation and soil bioremediation

**DOI:** 10.1016/j.scitotenv.2023.166067

**Published:** 2023-12-01

**Authors:** Dennis Beesigamukama, Chrysantus M. Tanga, Subramanian Sevgan, Sunday Ekesi, Segenet Kelemu

**Affiliations:** International Centre of Insect Physiology and Ecology, P. O. Box 30772-00100, Nairobi, Kenya

**Keywords:** Entomocomposting, Environmental cleanup, Nutrient recycling, Frass fertilizer, Soil health, Circular economy

## Abstract

The innovative use of insects to recycle low-value organic waste into value-added products such as food, feed and other products with a low ecological footprint has attracted rapid attention globally. The insect frass (a combination unconsumed substrate, faeces, and exuviae) contains substantial amounts of nutrients and beneficial microbes that could utilised as fertilizer. We analyse research trends and report on the production, nutrient quality, maturity and hygiene status of insect-composted organic fertilizer (ICOF) generated from different organic wastes, and their influence on soil fertility, pest and pathogen suppression, and crop productivity. Lastly, we discuss the impact of entomocomposting on greenhouse gas mitigation and provide critical analysis on the regulatory aspects of entomocomposting, and utilization and commercialisation ICOF products. This information should be critical to inform research and policy decisions aimed at developing and promoting appropriate standards and guidelines for quality production, sustainable utilization, and successful integration of entomocompost into existing fertilizer supply chains and cropping systems.

## Highlights

• Insects are fast and efficient recyclers of organic waste into quality fertilizer.
• Insect frass fertilizer contains higher nutrients than conventional fertilizers.
• Entomocomposting suppresses pathogens and reduces greenhouse gas emissions.
• Frass fertilizer boosts soil health, crop productivity and climate resilience.
• More research and enabling policies are required to improve entomocomposting.

## Graphical abstract

**Figure:**
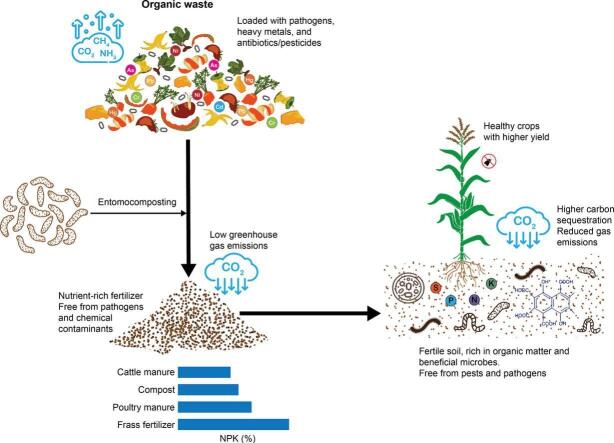

## 1 Introduction

Globally, urban areas generate more than two billion tonnes of decomposable organic waste per year. However, only about 16 % of these wastes is recycled, while >46 % is discarded (Kaza et al., 2018). In eastern Africa, organic waste accounts for 65–77 % of solid waste collected (Okot-Okumu, 2012). Due to the increasing population in sub-Saharan Africa (SSA), annual bio-waste generation is expected to increase by >3 folds, from 175 to 516 million tonnes by 2050 (Kaza et al., 2018). Yet, only 4 % is recycled and the rest is left to degrade in the open, threatening environmental and human health (Aryampa et al., 2021; Nweke and Sanders, 2009). Furthermore, landfilling disrupts biogeochemical cycles because it locks nutrients that would have been recycled for agricultural use (Amoding, 2007). There is, therefore, an urgent need to effectively recycle organic waste to conserve the environment and for economic empowerment.

The utilization of entomocomposting to convert organic waste into food, feed, fertilizer among other products, is a concept of circular economy that has attracted global attention (Beesigamukama et al., 2021a, Beesigamukama et al., 2021b; Bortolini et al., 2020; Houben et al., 2020; Makkar et al., 2014; Moruzzo et al., 2021; Poveda et al., 2019; Song et al., 2021; van Huis, 2013). Saprophytic insects such as black soldier fly (*Hermertia illucens* L.) (BSF) have high potential to convert organic waste into protein-rich larval insect biomass for feed and organic fertilizer for soil health management (Chia et al., 2019; Schiavone et al., 2017; van Huis and Oonincx, 2017; Al-Qazzaz et al., 2016; Rana et al., 2015; Caruso et al., 2014; van Huis, 2013; St-Hilaire et al., 2007).

**Fig. 1:**
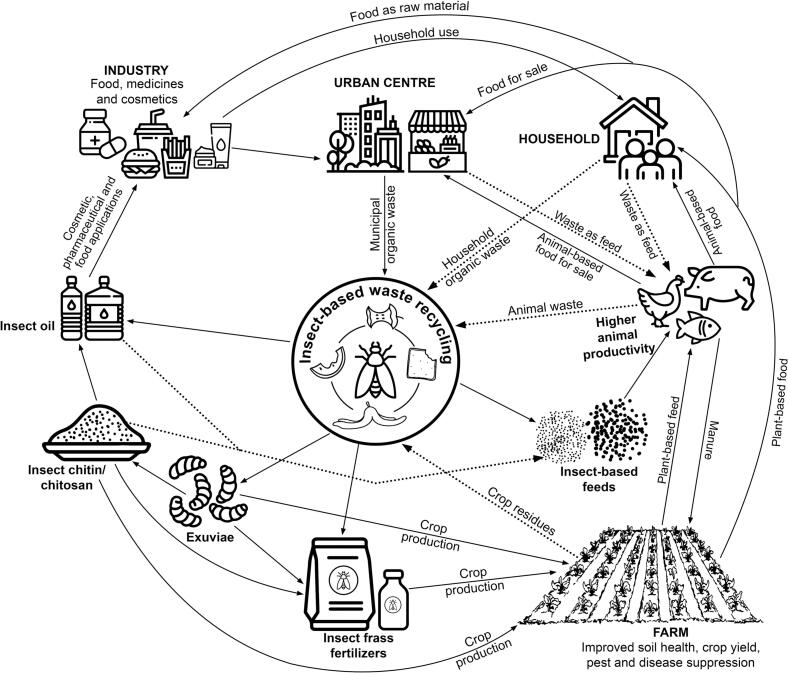
Circular economic model of insect farming.

Until recently, research on edible insect farming has mostly focused on feed and food production, and little has been done to explore other transformative products, especially the insect-composted organic fertilizer (ICOF) component. Yet, integrating insect protein with organic fertilizer could go a long in improving the profitability of edible insect farming for farmers already engaged in the production of insect-based food and feed ingredients, through circular economy (Fig. 1) (Beesigamukama et al., 2021a; Chen et al., 2019a, Chen et al., 2019b). The organic fertilizer generated would address soil health challenges to crop production and food security in low and middle income countries (Cobo et al., 2010; FAO, 2017; Stewart et al., 2020; Tully et al., 2015; Wortmann et al., 2019). Recent estimates projects a worldwide ICOF market value of USD 319.7 million by 2029 (Meticulous Market Research Pvt. Ltd.). Indeed, ICOF technologies strongly contribute to several development strategies aligned to sustainable development goals (SDGs), including SDG 1 (no poverty), SDG 2 (zero hunger), SDG 3 (good health and well-being), SDG 5 (gender equality), SDG 6 (clean water and sanitation), SDG 8 (decent work and economic growth), SDG 11 (sustainable cities and communities), SDG 12 (responsible consumption and production), SDG 13 (climate action), and SDG 15 (life on land).

Insect-composted organic fertilizer which is synonymously referred to as entomocompost is an organic fertilizer derived from insect frass (a by-product combination of unconsumed substrate, faeces, and exuviae from larval meal production) (Beesigamukama et al., 2021b). It has sufficient nutrients (Lalander et al., 2015; Oonincx et al., 2015) that have been harnessed as fertilizer (Houben et al., 2020, Houben et al., 2021; Menino et al., 2021; Beesigamukama et al., 2020c; Klammsteiner et al., 2020; Poveda et al., 2019).

Saprophytic insects such as BSF can suppress pathogens (Lalander et al., 2013, Lalander et al., 2015; Erickson et al., 2004), and attenuate chemical pollutants (Lalander et al., 2016; Diener et al., 2015) contained in organic wastes. This is because insects secrete antimicrobial peptides (Park et al., 2014; Zdybicka-Barabas et al., 2017) and digestive enzymes (Kim et al., 2011) that been found effective in minimising pathogens and chemical pollutants. The high rate of waste degradation by BSF larvae (40–80 %) (Diener et al., 2009, Diener et al., 2011) enables them to recycle organic wastes into organic fertilizers (Choi and Hassanzadeh, 2019a, Choi and Hassanzadeh, 2019b; Lalander et al., 2015, Lalander et al., 2018; Oonincx et al., 2015) within a shorter period (≤ 5 weeks) compared to ordinary composting methods (8–24 weeks) (Beesigamukama et al., 2021b). The innovation could clearly benefit a large population of farmers within a short period of time by supplying high-quality and affordable organic fertilizers.

Insect growth performance and the quality of ICOF generated are greatly influenced by the source and quality of rearing substrates (Arabzadeh et al., 2022; Lalander et al., 2019; Chia et al., 2018a; Oonincx et al., 2015). Organic wastes with low nutrients and high levels of chemical pollutants hinder insect growth (Cammack and Tomberlin, 2017; Diener et al., 2015), and produce frass with low nutrients (Oonincx et al., 2015). Thus, the quality of ICOF generated, and its impact on crop productivity, soil health, greenhouse gas mitigation, pests and pathogen suppression largely depend on the physical-chemical characteristics of rearing substrates, conditions in the rearing unit, bioconversion time, and presence or absence of frass treatment.

Knowledge on the nutrient quality, maturity and hygiene status of ICOF generated by different saprophytic insects and their influence on soil health, crop growth, yield and nutritional quality is scattered. Therefore, this review aimed at providing comprehensive analysis of entomocomposting with emphasis on nutrient dynamics, maturity, stability, and biological and chemical contaminants of ICOF generated from composting of organic waste using different insect saprophytic insects. The paper analyses current research on the influence of ICOF on soil fertility, nutrient release and crop productivity, and provides key recommendations for optimal utilization of insects for organic fertilizer production, and application of ICOF for sustainable soil health management.

## 2 Research trends in insect-composted organic fertilizer

**Fig. 2:**
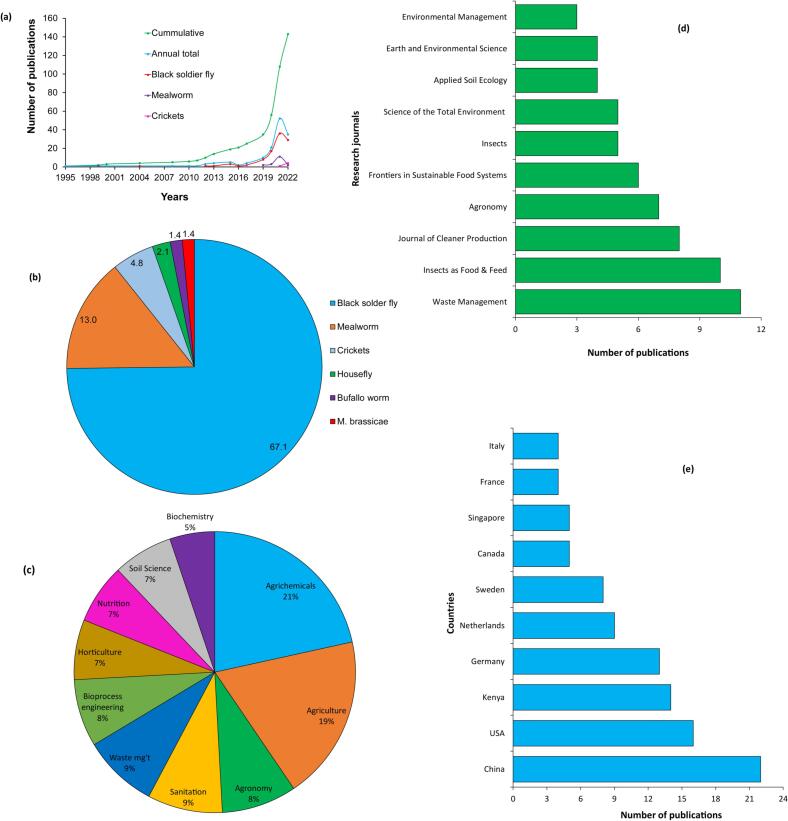
Trends in insect-composted organic fertilizer publication outputs (a), distribution of publications among insect species (b), key research concepts (c), top ten research journals (d) and top ten countries in insect-composted organic fertilizer research (e).

There has been an increase in publications focusing on the production, quality control and application of ICOF for soil health management. The web of science (https://www-webofscience-com) and google scholar search engines showed >140 publications on ICOF from 1995 to December 2022 (Fig. 2a), with a significant increase in research outputs observed from 2017 and beyond. This indicates a shift towards cleaner production methods and circular economy, and the pursuit for sustainable alternative inputs for agricultural production, which is a key step towards the realization of sustainable development goals (Barragan-Fonseca et al., 2020). The largest fraction of publications (67 %) have focused on the black soldier fly frass fertilizer followed by mealworm (13 %) and cricket frass fertilizers (4.8 %) (Fig. 2b). The dominance of black soldier fly research could be largely attributed to its higher reproduction rate, efficiency of bio-waste conversion, shorter developmental time, and lower production costs, compared to other insects most research has focused on concepts such as agrichemicals, agriculture, waste management, sanitation, agronomy, soil science, among others (Fig. 2c), highlighting the critical role of insect-based technologies in improving agricultural productivity, environmental health, and mitigating climate change.

**Fig. 3:**
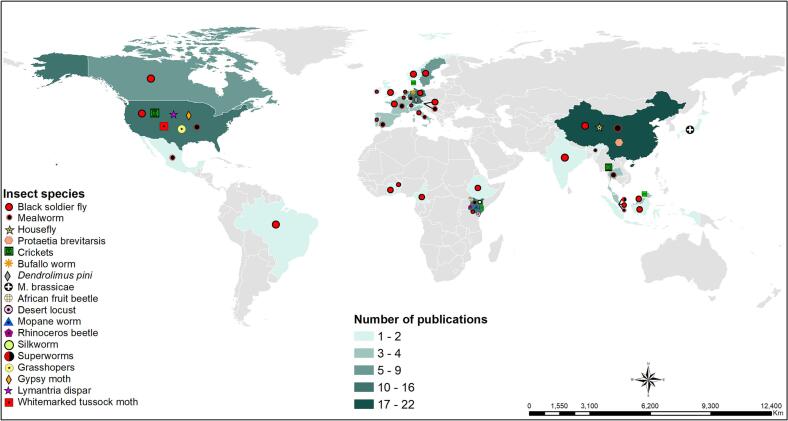
Global distribution of research outputs on insect-composted organic fertilizer and insect species used in generation of organic fertilizer.

Research outputs on ICOF have mostly been published in journals whose scopes focus on agriculture and environmental science (Fig. 2d). Waste Management, Journal of Insects as Food and Feed, Cleaner Production and Agronomy are among the journals leading the publication of research outputs on ICOF (Fig. 2d). The emergence of new journals that are solely focused on edible insect farming innovations and revision of scopes by existing journals to include edible insects indicate high global interest in the dissemination of insect-based technologies. In terms of global share of research outputs, China, USA and Kenya are the top three publishers, but Europe has more countries in the top ten list (Figs. 2e, 3). Kenya is the only African country with the highest record of publication on ICOF research followed by Benin and Ghana with two and one publications, respectively. The increase in publications, research concepts and global spread of research outputs on frass fertilizer highlight the growing interest in the use of ICOF to address the challenges of soil degradation, food insecurity, environmental degradation and unemployment at a global scale.

The low number of publications emanating from Africa and South America which are among the contents with the highest diversity of edible insects (Kelemu et al., 2015), highlights the need for investment in edible insect technologies. On the other hand, the low research outputs from most parts of Asia and Australia's lack of research publication on edible insects could be largely attributed to limited interest insect consumption due to cultural norms. However, it is anticipated that the emergence of novel products such as insect-based biodiesel and therapeutics (Rehman et al., 2018) will increase research interest and commercialization of insect-based technologies in these continents.

## 3 The insect-assisted composting (entomocomposting) process

As opposed to conventional composting which is solely mediated by microbes (fungi and bacteria), entomocomposting involves the breakdown of bio-wastes by insect larvae and microbes under aerobic conditions for a specific period to produce insect larval biomass and frass. The entomocomposting process starts immediately after egg hatching, when the newly hatched larvae (neonates) descend into the substrates and start to feed. Larval feeding can be *ad libitum* or lump sum, whereby lump sum feeding has been found more effective (Dortmans, 2015). For instance, in black soldier fly (BSF), the bioconversion process takes place during the larval stages of growth and ranges between 8 and 70 days, depending on the substrate quality and rearing conditions (Chia et al., 2018a; Liu et al., 2018; Manurung et al., 2016; Supriyatna et al., 2016). The frass is obtained at the end of the bioconversion process (when the larvae reach prepupae stage), by sieving to separate it from the larvae (Beesigamukama et al., 2021b). For every tonne of BSF larvae, 10–34 t insect-composted organic fertilizer can be obtained (Beesigamukama et al., 2021a). The quantity of frass obtained varies depending on the length of the bioconversion process and quality of the rearing substrate. Studies have shown that wastes with high recalcitrant carbon tend to reduce the degradation efficiency of BSF larvae and produce higher quantities of frass (Beesigamukama et al., 2020b, Beesigamukama et al., 2021b).

### 3.1 Optimizing entomocomposting

**Fig. 4:**
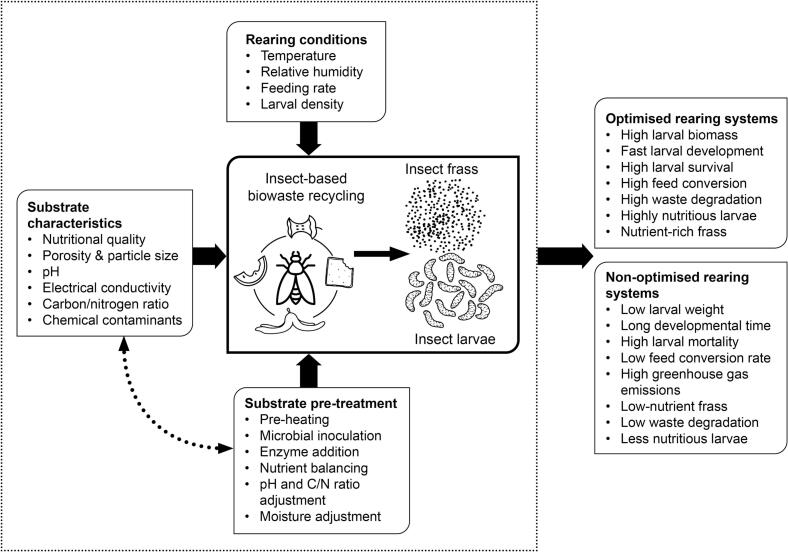
Illustration of factors influencing entomocomposting and insect bioconversion processes.

The length of the bioconversion process, waste degradation efficiency and quantity of frass obtained largely depend on the waste type, nutritional value (Lalander et al., 2020; Isibika et al., 2019; Liu et al., 2018), pH (Ma et al., 2018), larval feeding rate, larval density, rearing temperatures, particle size and other physical-chemical characteristics of the feedstock (Beesigamukama et al., 2020b; Parodi et al., 2020; Chia et al., 2018b; Supriyatna et al., 2016; Diener et al., 2009) (Table 1, Fig. 4). Easily digestible waste result in high BSF larval biomass and high waste degradation efficiencies, while substrates with poor nutritional quality, high C/N ratio, electrical conductivity, and extremes of pH and moisture mostly slowdown the bioconversion process (Beesigamukama et al., 2020b; Chen et al., 2019a, Chen et al., 2019b; Ma et al., 2018).

**Table 1:** Bioconversion performance of insect larvae reared on different substrates.

Substrate	Age of larvae (days)	Feeding rate (mg larva^−1^ day^−1^)	Larval density	Waste degradation (%)	BCR (%)	Larval development time (days)	Larval weight (g larva^−1^)	Larval survival rate (%)	Reference
Bran and corn flour	6	75–111	ND	ND	ND	21–29	0.16–0.2	89–99	Ma et al. (2018)
Alfalfa, wheat bran and corn meal	5	80	0.06 larvae cm^−3^	59–61	ND	10	0.14–0.15	94–96.5	Meneguz et al. (2018)
Brewer's waste pig manure and grass	7	200	0.43 larvae cm^−3^	14–39	1.9–5.4	15–70	0.013–0.059	89–98	Liu et al. (2018)
Banana peels	10	40	0.6 g cm^−2^	9–70	0.9–15	30	0.03–0.23	51–100	Isibika et al. (2019)
Food waste	5	30	2 larvae cm^−2^	43–52	18–28	14	0.29–0.31	67–87	(Ermolaev et al., 2019)
Household food waste	ND	200	1.5 larvae cm^−2^	35–69	18–33	14–15	0.1–0.37	19–97	(Lindberg et al. (2022)
Okara (soy bean pulp)	5	133	6.5 larvae cm^−3^	85.7 %	10.17	17			

ND = not determined.

Although some studies (Wu et al., 2020; Diener et al., 2015) have reported little or no impacts of heavy metals on insect growth, weight gain, bioconversion performance and sex ratio, others (Cai et al., 2018) have associated larval mortality and decreased larval weight gain to high heavy concentrations. However, the bioaccumulation of heavy metals in insect tissues varies with specific metal elements. For example, feedstock with high concentrations of cadmium and copper has been found to increase accumulation of two metals in insect tissues (Wu et al., 2020; Diener et al., 2015). On the contrary, the accumulation of lead and zinc is usually low even when insects are fed on substrates with excess levels of the two heavy metals (Diener et al., 2015).

High exposure to heavy metals induces significant shifts in insect microbiome and drastic reduction in the gut bacterial diversity (Wu et al., 2020); this could have deleterious impacts on insect immunity, physiology and ecological fitness. While exposure to heavy metals increases accumulation in insect biomass, the oil extracted from such insects has been found free of heavy metals (Cai et al., 2018), indicating tolerance of insects to heavy metal contamination and high suitability for recycling contaminated wastes.

Moisture content and ventilation of the feedstock also affect BSF larval feeding, survival, and weight gain (Lalander et al., 2020). For example, substrates with moisture content of 75–76 % result in high larval biomass and survival rate, while moisture levels below 65 % or above 75 % cause significant reduction in survival rate, weight gain, and efficiency of waste biotransformation (Lalander et al., 2020; Chen et al., 2019a, Chen et al., 2019b).

The inclusion of biochar in feedstock for BSF rearing has been found to improve the retention of moisture and nutrients, thus increasing larval biomass (Beesigamukama et al., 2020b). For substrates with moisture of 80–90 %, such as fruits, vegetables and food waste, the composting efficiency of BSFL can be increased by improving ventilation during the process through moisture removal, and regulation of temperature and relative humidity within the substrates (Lalander et al., 2020). Although the pH of the feedstock varies highly during the bioconversion process, the favourable pH for optimal BSFL performance is 6–7 (Ma et al., 2018), especially at the initial stages (Meneguz et al., 2018). Isibika et al. (2019) reported higher composting efficiency of BSFL reared on banana peels after adjusting substrate pH using sulphuric acid.

Practices such as increasing the frequency of feeding significantly have been found to increase the efficiency of waste bioconversion (1.5–2.2-folds), waste degradation and reduce moisture content of the BSF frass (Zhang et al., 2021). Inoculation of rearing substrates with beneficial bacteria (*Arthrobacter* AK19, *Bifidobacterium breve*, *Arthrobacter* AK19 and *Rhodococcus rhodochrous* 21,198) is also critical in accelerating waste degradation and entomocomposting by BSFL (Kooienga et al., 2020). Furthermore, application of lignin-digesting microorganisms has been recommended to improve the bioconversion efficiency of lignin-rich substrates by insect larvae (Liu et al., 2018). Addition of enzymes such as cellulases, ß-glucosidases and hemicellulases in a mixture of cabbage and lettuce wastes at the initial stages can increase bioconversion and waste degradation efficiencies of BSFL by 22 % and 14 %, respectively (Lindberg et al., 2022). On the other hand, pre-treatment of banana peels through microbial inoculation (fungi and bacteria) and addition of non-protein nitrogen can enhance the digestibility, nutrient availability, and composting efficiency of BSFL (Isibika et al., 2019). However, the same study found that heat treatment at 120 °C reduced BSFL performance due to increased levels of tannins and phenolic compounds.

### 3.2 Insect frass composting

The frass obtained from BSFL farming may be subjected to further composting to improve the level of maturity and stability for field application as fertilizer. Beesigamukama et al. (2021b) found a composting period of 5 weeks sufficient to transform BSF frass into quality organic fertilizer, while Anyega et al., 2021a, Anyega et al., 2021b required 16 weeks to compost BSF frass made of rice husks and spent grain into organic fertilizer. Therefore, composting of frass is an essential step towards increasing mineralization rate, reducing phytotoxicity and elimination of pathogens. In insect frass, nitrogen (N) is mostly present in ammonium form (Green and Popa, 2012), yet high ammonium can cause toxicity to plants (Bernal et al., 1998; Wong, 1985; Wong et al., 1983). Thus, composting is essential to improve nitrification rates after soil application. However, the frass can be directly used for crop production (Gärttling et al., 2020; Klammsteiner et al., 2020), although this poses a risk of nutrient immobilization, especially if the levels of C/N ratio, ammonium/nitrate ratio are beyond the recommended thresholds (Bernal et al., 1998, Bernal et al., 2017).

Composting also influences the nutrient levels of ICOF, with compost quality highly dependent on the composting method. Aerobic composting of Okara and wheat bran frass decreased the concentrations of N and phosphorus (P) by 33 % and 27 %, respectively, while natural composting of the same frass increased total N and P by 26 % and 32 %, respectively (Song et al., 2021). However, the same study found that aerobic composting increased nitrification by 17 folds while natural composting reduced nitrate concentration by 6.3 times. It was further noted that composting the frass using both methods decreased the concentrations of potassium (K), calcium (Ca), and micronutrients, with higher losses observed under aerobic composting (Song et al., 2021).

### 3.3 Physical-chemical changes during entomocomposting

It is crucial to evaluate the maturity and stability status of ICOF to determine whether the fertilizer produced is free of toxic substances that could inhibit seed germination and plant growth (Bernal et al., 1998, Bernal et al., 2009, Bernal et al., 2017; Guo et al., 2012; Teresa and Remigio, 2011; Goyal et al., 2005). Compost maturity is assessed using several physical-chemical (Table 2) and biological parameters. However, the concept of maturity and stability of insect frass fertilizer has received limited research attention, yet commercialization of insect frass fertilizer requires approval from regulatory bodies, based on the threshold values set for various maturity parameters. It should be noted that the level of maturity and stability of ICOF largely depends on the insect species used in bioconversion, bioconversion time, and type and quality of the rearing substrate.

**Table 2:** Maturity and stability status of ICOF derived from different wastes in comparison with recommended threshold values for mature compost.

Observed values	Reference	Recommended value for mature compost
pH		
6.8–7.4	Chen et al., 2019a, Chen et al., 2019b	6–8 (Bernal et al., 2009)
7.2–8.8	Palma et al. (2020)	
7.3	Anyega et al., 2021a, Anyega et al., 2021b	
6.6–7.8	Beesigamukama et al. (2020b)	
6.9–7.6	Beesigamukama et al. (2021b)	
7.2–9.0	Liu et al. (2022)	
8.8	Setti et al. (2019)	
7.4	Visvini et al. (2022)	
6.9	Fuhrmann et al. (2022)	

Electrical conductivity (mS cm^−1^)		
2.9–4.5	Chen et al., 2019a, Chen et al., 2019b	<4 mS cm^−1^ (Huang et al., 2004)
0.36–4	Beesigamukama et al. (2020b)	
0.7–2.7	Beesigamukama et al. (2021b)	
8.5	Setti et al. (2019)	
1.21	Visvini et al. (2022)	

Ammonium (mg kg^−1^)		
1000–9000	Chen et al., 2019a, Chen et al., 2019b	< 400 mg kg^−1^ (Bernal et al., 1998)
400–5695	Palma et al. (2020)	
588–4516	Beesigamukama et al. (2020b)	
25–3466	Beesigamukama et al. (2021b)	
8870.2	Visvini et al. (2022)	
4667.3	Fuhrmann et al. (2022)	

Ammonium/nitrate ratio		
0.3–34.1	Beesigamukama et al. (2021b)	≤ 0.16 (Bernal et al., 1998)
1.63	Visvini et al. (2022)	
20–75	Chen et al., 2019a, Chen et al., 2019b	< 4 mg kg^−1^ (Bernal et al., 1998)
1.0–1.7	Beesigamukama et al. (2021b)	
18.7	Fuhrmann et al. (2022)	

C/N ratio		
22–38	Palma et al. (2020)	< 12 (Bernal et al., 1998)
10.7	Anyega et al., 2021a, Anyega et al., 2021b	
12.8–20.3	Beesigamukama et al. (2020b)	
10.5–23.4	Beesigamukama et al. (2021b)	
7.0–9.6	Song et al. (2021)	
8.8	Setti et al. (2019)	
16	Visvini et al. (2022)	
12.7	Fuhrmann et al. (2022)	

Germination index (%)		
0–53	Palma et al. (2020)	< 80 % (Emino and Warman, 2004; Teresa and Remigio, 2011)
86	Anyega et al., 2021a, Anyega et al., 2021b	
56–274	Beesigamukama et al. (2020b)	
62–191	Beesigamukama et al. (2021b)	
73–168	Song et al. (2021)	
117–222	Setti et al. (2019)	
95–100	Visvini et al. (2022)	
14–69 %	Liang et al. (2022)	

Assessment of entomocomposts generated by nine edible insect species revealed that the BSFFF had a higher degree of compost maturity compared to ICOF produced by other insects, highlighting the influence of insect species, bioconversion time and rearing substrate on ICOF quality (Beesigamukama et al., 2022). Existing information shows that the values of pH and electrical conductivity of ICOF derived from various organic wastes are within the values recommended for mature compost (Table 2). However, frass derived from food waste, refined animal feed, and those amended with inorganic additives such as gypsum tend to have higher electrical conductivity (Setti et al., 2019; Tan et al., 2021). Application of ICOF with high electrical conductivity and pathogen loads can suppress plant growth, reduce crop yields, enhance the spread of crop diseases, and pollute the environment (Dzepe et al., 2022; Tan et al., 2021). The generally higher values of pH in BSF frass compared to conventional composts could be largely due to higher ammonium concentration (Green and Popa, 2012).

Insect-based composting has been found to improve compost maturity by accelerating degradation of dissolved organic matter fractions (carboxylic, alcohol and aliphatic components), and increase humification levels of ICOF derived from cattle manure, pig manure and chicken manure (Wang et al., 2021). Assessment of frass C/N ratio has shown that BSFL composting can significantly reduce the C/N ratio to values recommended for field application (〈12). The low C/N ratio of BSF frass is critical for ensuring higher nutrient mineralization and better synchrony for plant uptake (Beesigamukama et al., 2020c, Beesigamukama et al., 2021c; Adin Yéton et al., 2019).

Composting substrates with high lignin and cellulose such as sawdust, biochar, maize stalks and almond hulls produces frass with higher C/N ratio (Beesigamukama et al., 2020b, Beesigamukama et al., 2021b; Palma et al., 2020). Conversely, the inclusion of small quantities of rice straw (1–2 % *w*/w) to chicken and borne meal feedstock improves organic matter degradation in terms of humification and aromatization degrees of BSF frass, but inclusion levels of ≥3 % are undesirable (Liang et al., 2022). Moreover, insects such as *Protaetia brevitarsis* have ability to efficiently breakdown lignin-rich substrates, and improve compost maturity and humification indices (Wei et al., 2020). Although organic fertilizers with higher C/N ratio could induce nutrient immobilization, they are associated with the benefits of boosting soil carbon and organic matter stocks that are critical for sustainable health management (Musinguzi et al., 2016; Vanlauwe et al., 2014, Vanlauwe et al., 2015).

The ammonium concentration and ammonium/nitrate ratios of most ICOF are usually above the threshold values of <400 mg kg^−1^ and ≤ 0.16, respectively (Table 2), largely because insect frass contains a lot of organically bound ammonium, and dissimilatory reduction of nitrate to ammonium by insect larvae (Green and Popa, 2012). Composts with excessive ammonium and salt levels have been associated with increased phytotoxicity (Teresa and Remigio, 2011), warranting further treatment of frass to improve maturity and stability. Moreover, previous studies have demonstrated that the accelerated composting phase by saprophytic insects cannot eliminate all the phytotoxins, especially phenols, and recommended an additional composting period of 5–8 weeks to improve seed germination rates and germination index (Liang et al., 2022; Song et al., 2021; Beesigamukama et al., 2021b; Beesigamukama et al., 2020b). The chitin and high ammonium contained in BSF frass fertilizer could be responsible for low values of seed germination index observed in uncomposted frass (Palma et al., 2020). Composting BSF frass derived from a mixture of okra and wheat bran has been found to significantly increase compost maturity, germination rate and germination index of *Brassica rapa* and cabbage seeds (Song et al., 2021).

It should be noted that up to date, the maturity and stability of all ICOF are assessed based on indices and critical values established for conventionally composted organic fertilizers (Table 2). This could be inaccurate due to the obvious differences between fly larval composting and conventional composting in terms of the time taken, composting temperatures, microbiota, pathogen suppression and quality of substrate used. Therefore, there is urgent need to determine the critical physical, chemical and biological indicators, and to establish the threshold values that could be used to assess the maturity and stability of ICOF generated by different saprophytic insects.

## 4 Nutrient quality of insect-composted organic fertilizer

It has been demonstrated that ICOF contains adequate quantities of all the nutrients required for optimal crop growth (Anyega et al., 2021a, Anyega et al., 2021b; Beesigamukama et al., 2021b; Song et al., 2021; Tanga et al., 2021a, Tanga et al., 2021b, Tanga et al., 2021c; Beesigamukama et al., 2020b; Gärttling et al., 2020; Setti et al., 2020). Comparative assessment of ICOF generated by nine insect species revealed adequate concentrations of all plant nutrients and nutrient supply capacities, but there were great variations across insect species (Beesigamukama et al., 2022). For example, the BSF frass fertilizer had the highest concentrations of N and K while cricket frass fertilizer was superior in P (Beesigamukama et al., 2022).

**Fig. 5:**
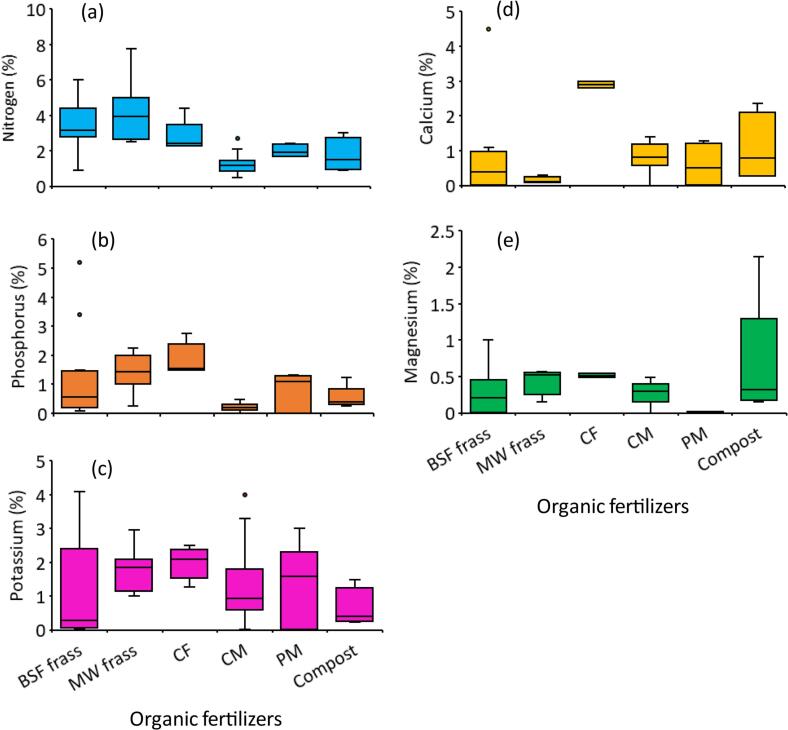
Concentrations of macronutrients (a – c) and secondary nutrients (d – e) in insect-composted organic fertilizers and commonly used organic fertilizers. BSF frass = black soldier fly frass fertilizer MW frass = mealworm frass fertilizer, *CF* = cricket frass fertilizer, CM = cattle manure, PM = poultry manure, compost = conventional organic fertilizers from a mixture of crop and animal wastes.

The concentrations of macronutrients and secondary nutrients in ICOF are usually higher than those of conventionally composted manures such as cattle dung, poultry manure and compost from a mixture of crop and animal wastes (Fig. 5) (Tumuhairwe et al., 2009). The concentrations of N, P and K in ICOF range from 2 to 6 %, 0.1–5 %, and 0.05–4 %, respectively, and are highly influenced by type of organic waste used for insect rearing (Supplementary Table 1). The high N in ICOF could be due to the high efficiency of nutrient conservation associated with entomocomposting, mainly due to low (mesophilic) composting temperatures, absence or minimal losses through leaching and denitrification (Beesigamukama et al., 2021b). Also, the beneficial bacteria of phyla *Proteobacteria, Firmicutes* and *Bacteroidetes* and genera *Enterococcus, Providencia* and *Morganella* found in insect larval guts have been found crucial in improving nutrient transformation, thus producing ICOF with higher nutrient concentrations than the raw organic substrates (Ao et al., 2021).

Nutrient concentrations in frass fertilizer largely depend on the initial concentrations in the rearing substrates, substrate moisture content and C/N ratio, presence, or absence of nutrient conservation strategies during composting and bioconversion time. Nitrogen is the most important nutrient for BSFL growth because it is a major constituent of protein which BSFL are known to supply in feeds. Nitrogen undergoes several dynamic changes during entomocomposting; the major pathways include larval uptake, volatilisation and denitrification (Chen et al., 2019a, Chen et al., 2019b; Ermolaev et al., 2019; Oonincx et al., 2015). Because the BSFL are used in feed formulation, the N accumulated in larval biomass is not considered a loss. Conversely, most N losses during BSFL composting occur in terms of volatilisation. For example, Oonincx et al. (2015) and Lalander et al. (2015) reported N losses of 23–78 % and 44 %, respectively through ammonia volatilisation.

**Source:**Beesigamukama et al., 2020a, Beesigamukama et al., 2020c, Beesigamukama et al., 2022, 2018), Fuhrmann et al. (2022), Liang et al. (2022), Visvini et al. (2022), Anyega et al., 2021a, Anyega et al., 2021b, Chiam et al. (2021), Ebanyat et al. (2021)Song et al. (2021), Tanga et al., 2021a, Tanga et al., 2021b, Tanga et al., 2021c, Watson et al. (2021a), Adekiya et al. (2020), Gärttling et al. (2020)Setti et al. (2019), Halloran et al. (2017), Mucheru-Muna et al., 2007, Mucheru-Muna et al., 2014, Ogwang et al. (2012), Amoding et al. (2011), Boateng et al. (2009), Mugwe et al. (2009), Tittonell et al. (2008), Rufino et al. (2007).

The BSFL perform optimally on substrates with neutral to alkaline pH and low C/N ratios (Ma et al., 2018). Yet, substrates with pH >7.5 and C/N ratios lower than 15 induce ammonia volatilisation (Chen et al., 2019a, Chen et al., 2019b; Sánchez et al., 2017; Bernal et al., 2009; Huang et al., 2004). Nitrogen conservation during BSFL composting can be ensured by regulation of moisture, adjustment of substrate C/N and pH. Substrates with moisture content higher than 75 % have been found to increase N retention in frass due to decrease in ammonia volatilisation during BSFL feeding (Chen et al., 2019a, Chen et al., 2019b). Also, adjusting C/N ratio of spent grain to 15 using sawdust has been found to increase the N content of BSF frass by up to 21 % compared to un-amended substrates (Beesigamukama et al., 2021b). This is because sawdust enhances the binding of ammonia onto phenolic compounds, thus reducing volatilisation (Lim et al., 2017). Likewise, substrate amendment with by inclusion of 5–15 % gypsum and 10–20 % biochar can significantly increase N retention during BSF rearing (Beesigamukama et al., 2020b), although gypsum rates above 5 % decreased larval performance.

In comparison to N, however, BSFL-mediated composting increased the concentrations of P in frass by up to 50 % (Oonincx et al., 2015). Similarly, Beesigamukama et al. (2020b) reported significant increases in K concentration of frass after composting a mixture of brewery spent grain and biochar using BSFL.

## 5 Chemical and microbial contaminants in insect-composted organic fertilizers

Some of the organic wastes (*e.g.*, animal manures and industrial wastes) used in insect rearing could be contaminated with heavy metals, antibiotics and pathogens (*Salmonella spp.*, *E. coli*, *Clostridium perfringens*, *Listeria monocytogenes and Enterococcus*) (Liu et al., 2017; Brochier et al., 2012) that may not be reduced to permissible levels by conventional composting methods (Brinton, 2000). The BSF larval gut has antimicrobial peptides such as StomoxynZH1 and DLP4 (Mudalungu et al., 2021; Vogel et al., 2018) that have potential to suppress several microbial pathogens that usually persist in compost (Vogel et al., 2018; Elhag et al., 2017; Yi et al., 2014). The other mechanisms of pathogen suppression include microbe ingestion and/or lysis inside the insect gut (Tanga et al., 2021b).

The BSF larvae have high efficiency to significantly reduce bacterial pathogens present in animal manure (*E. coli* and salmonella) to minimum levels (Erickson et al., 2004), thus improving the suitability of such manure for agricultural use and preventing environmental pollution. Entomocomposting a mixture of animal and human waste using BSF larvae suppressed *Salmonella spp.*, and reduce thermotolerant coliforms and viruses to permissible levels, but not for *Enterococcus spp.* (Lalander et al., 2013, Lalander et al., 2015). Furthermore, bioconversion of chicken and swine manure using *Protaetia brevitarsis* larvae has been found to significantly lower the numbers of antibiotic resistance genes and mobile genetic elements in resultant frass fertilizer within one week, thus a sustainable technique for removing antibiotic resistance genes in organic fertilizers (Zhao et al., 2022).

During BSF composting, higher pathogen deactivation has been registered at mesophillic temperatures (27–32 °C) and high pH (Erickson et al., 2004). Thus, growth media consisting insect frass have been found to be free from food-borne pathogens such as *Campylobacter, Listeria, Salmonella* and *Yersinia* (Tan et al., 2021). Heat treatment at 70 °C for 1 h was found efficient in eliminating most pathogenic microbes in insect frass, thus improving suitability for use as organic fertilizer (Van Looveren et al., 2022). However, prolonged heating may induce ammonia volatilisation and reduce the nutrient levels in frass, and may not improve maturity and stability parameters such as pH, ammonium concentration, ammonium/nitrate ratio, humification index, and C/N ratio as well as germination index. Thus, composting or fermentation remains the most appropriate treatment approaches for improving the maturity and stability of frass for fertilizer use (Beesigamukama et al., 2021b).

The BSFL can significantly reduce chemical contaminants in organic wastes without bioaccumulation of the target substances in the insect biomass (Bohm et al., 2022; Lalander et al., 2016). Lalander et al. (2016) found that BSFL accelerated the degradation of pesticides and pharmaceuticals contained in dog food, achieving half-lives of 0.6–1.9 days. Apart from biochemical reactions that transform pesticides/antibiotics into less harmful compounds, it is anticipated that reduction in chemical contaminants also takes places during moulting and through defaecation in form of frass (Bohm et al., 2022; Wu et al., 2020; Diener et al., 2015). Composting BSF frass from chicken, pig, and dairy manures mixed with cornstalk can enhance metal adsorption, and cause shifts in the microbial community structure with increased abundance of heavy metal resistance bacteria, consequently decreasing the concentrations of copper, zinc, lead and cadmium in the frass fertilizers generated (Liu et al., 2022).

Wastes with high concentration of cadmium and zinc (≥ 1000 mg kg^−1^) have been found to cause BSF larval mortality and increase bioaccumulation of heavy metals in larval biomass (Diener et al., 2011, Diener et al., 2015), reducing suitability for inclusion in animal feeds. Furthermore, frass from insects reared on wastes contaminated with heavy metals usually contains high levels of such metals (Wu et al., 2020; Diener et al., 2015), thus unsuitable for organic fertilizer production (Cai et al., 2020). It is worth noting that in the above studies, the wastes were spiked to achieve heavy metal concentrations that are abnormally higher than those found in most organic wastes used for BSF rearing. Thus, waste treatment using BSFL remains an efficient and low-cost technology for reducing the risk of spread of chemical contaminants in the environment, bioaccumulation and biomagnification when the frass fertilizer is used for food crop production.

## 6 Greenhouse gas emissions during insect frass composting

Recent studies have demonstrated that adoption of insect-based technologies for feed, oil and organic fertilizer production has potential to reduce carbon dioxide emissions by 55–83 % and depletion of conventional energy sources such as fossil fuel by 46 %, indicating higher environmental sustainability and low ecological footprint (Van PhI et al., 2020). Entomocomposting is more sustainable than conventional composting and incineration due to less greenhouse gas (GHG) emissions (Chen et al., 2019b; Mertenat et al., 2019; Halloran et al., 2017). Waste treatment using BSFL composting has been found to reduce GHG by up to 99 % compared to conventional composting (Chen et al., 2019a), as well as suppressing odorous emissions that would become a public nuisance and health hazard. For example, the direct GHG emissions attributed to BSF farming is 17 g CO_2_ equivalent per kg of dry larvae produced (Ermolaev et al., 2019) and 380 g CO_2_ equivalent per tonne of waste recycled (Parodi et al., 2020). Moreover, BSFL composting has been found to lower CO_2_ equivalence emissions by 47 times compared to conventional composting (Mertenat et al., 2019).

Using BSFL to compost a mixture of pig manure and corncob reduced methane (CH_4_), nitrous oxide (N_2_O) and ammonia (NH_3_) emissions by 73–100 %, 100 % and 82–90 %, respectively, compared to conventional composting, although the carbon dioxide (CO_2_) emissions increased due to continuous organic matter degradation by microbes in BSF larval gut (Chen et al., 2019a, Chen et al., 2019b). Furthermore, BSFL composting of food waste using significantly reduced emissions of CH_4_ and N_2_O compared to conventional composting, while NH_3_ was lowered to below detection limit (Ermolaev et al., 2019). The reduction in GHG emissions could be attributed to maximum oxygen circulation provided by continuous turning of BSFL during feeding that prevents development of anaerobic conditions. Furthermore, the low composting temperatures (mesophilic) associated with BSFL composting minimises greenhouse gas emissions (Beesigamukama et al., 2021b).

The quantity of greenhouse gases emitted during BSFL composting largely depends on temperature, pH, substrate characteristics (moisture content, C/N ratio and nutritional quality), bioconversion time, number of feeding times, and presence or absence of waste pre-treatment. Pre-treatment of food waste with bacterial cultures extracted from BSFL gut has been found to be more efficient at reducing GHGs than using BSFL alone (Ermolaev et al., 2019). Contrarily, substrates with high pH levels and moisture content of ≤65 % or ≥ 75 % emit higher amounts of greenhouse gases, especially NH_3_ and N_2_O (Song et al., 2021; Chen et al., 2019a, Chen et al., 2019b). Furthermore, higher bioconversion temperatures have been associated with increase in GHG emissions, especially CO_2_ and NH_3_ (Parodi et al., 2020). Increasing the number of feeding times also elevates greenhouse gas emissions (CO_2,_ N_2_O, CH_4_, and NH_3_) and carbon dioxide equivalence from BSF-mediated waste bioconversion systems, compared to one time feeding (Zhang et al., 2021).

Further composting of BSF frass has been reported to increase GHG emissions by 20–52 %, thus reducing the benefit of decreased greenhouse gas emissions (Song et al., 2021). It has been found that additional composting of frass and electricity requirements of BSF rearing contribute 69 % and 55 % of global warming potential from BSF farming (Mertenat et al., 2019). However, these values could be less in regions where farmers rely on solar energy to provide heat and dry BSF larvae, especially in the tropics. Nevertheless, it is prudent that frass composting should involve strategies for controlling GHG emissions, especially adjustment of substrate C/N ratio using carbon rich materials (sawdust, cereal straws, rice husks, biochar), pH adjustment and moisture balancing (Anyega et al., 2021a, Anyega et al., 2021b; Beesigamukama et al., 2020b, Beesigamukama et al., 2021b; Chen et al., 2019b).

## 7 Benefits of insect-composted organic fertilizers on soil quality and productivity

**Fig. 6:**
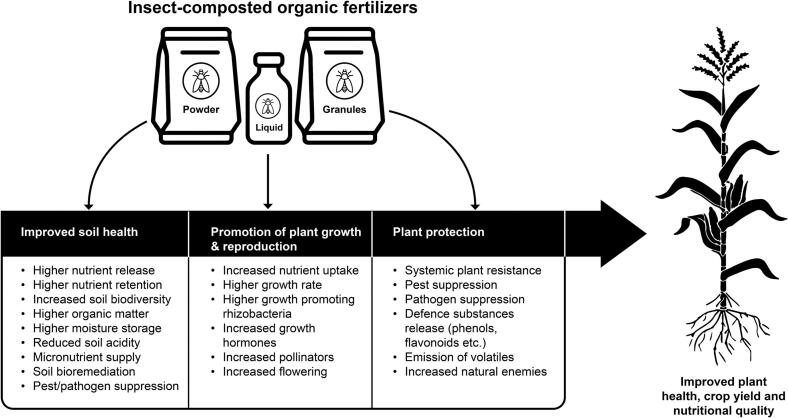
Benefits of entomocompost on soil health and crop productivity.

The benefits of ICOF on soil nutrient availability, nitrogen mineralization and synchrony for crop uptake, pests and disease suppression have been widely demonstrated (Fig. 6). Entomocompost is superior to compost and synthetic fertilizers in boosting populations of beneficial soil microbiota, reducing soil acidity, increasing nutrient availability for plant uptake (Beesigamukama et al., 2020a, Beesigamukama et al., 2020c, Beesigamukama et al., 2021c; Rummel et al., 2021). Moreover, soil amendment with 2–8 % of BSF frass fertilizer has been found to cause remarkable improvement in pH, organic matter, macronutrients, and fixation of heavy metals such as cadmium and lead by 8–57 % and 22–166 %, respectively (Wang et al., 2022).

Soil amendment with ICOF boosts organic matter and reduces GHG emissions, thus contributing to mitigation of global warming and adaptation to climate change (Gebremikael et al., 2022; Rummel et al., 2021; Song et al., 2021). However, recent studies have reported significantly higher carbon dioxide emissions from soils amended with BSFFF compared to conventional compost and unfertilized soil, due to increase in basal respiration (Fuhrmann et al., 2022); this warrants further research to generate accurate conclusions on the influence of ICOF on soil greenhouse emissions. Nevertheless, bioturbation activities of BSF larvae have been found beneficial in incorporating soil organic matter and increasing soil volume (Orozco-Ortiz et al., 2021). Moreover, insect frass has been found beneficial in accelerating litter decomposition, soil organic matter transformation, and enzymatic activities, and regulating nutrient cycling in natural ecosystems, which are critical for maintaining the soil food web and ecosystem balance (Hwang et al., 2022; Madritch et al., 2007). Because the N in frass is mostly in the form of ammonium, ICOF could act as a nature-based strategy for inhibiting nitrification, and thus increase soil N availability, reduce volatilisation, and adapt crop production to the shocks of climate change (Subbarao and Searchinger, 2021; Subbarao et al., 2009, Subbarao et al., 2012).

The influence of ICOF on soil quality is determined by fertilizer quality (nutrients, maturity and stability), rates of application and nutrient release, application method and time of application (Beesigamukama et al., 2020b; Poveda et al., 2019; Kagata and Ohgushi, 2012). Application of immature and unstable ICOF with low nutrients induces nutrient immobilization and hampers synchrony for crop growth (Kagata and Ohgushi, 2012). Supplementary application of synthetic fertilizers has been recommended to address the challenge of nutrient immobilization associated with ICOF, and synchronise mineralization with plant nutrient demands (Menino et al., 2022; Beesigamukama et al., 2021a; Gebremikael et al., 2020). Moreover, urease inhibitors have been found effective in regulating N mineralization from ICOF, thus reducing N loss and improving use efficiency (Watson et al., 2021b).

Application of ICOF has been found to improve biological soil fertility. Application of mealworm frass fertilizer boosts the microbiome responsible for soil nutrient cycling, especially bacteria and fungi responsible for solubilization of P, K and micronutrients, N fixing and proteolytic bacteria (Agustiyani et al., 2021; Watson et al., 2021b; Poveda et al., 2019), and consequently improved soil microbial activity and nutrient availability (Houben et al., 2020). Entomocompost increases the abundance and diversity of beneficial microbiota, especially those belonging to genera *Proteobacteria, Bacteroidetes, Terrimonas, Bacillus* and *Mortierella* that aid in N cycling, putrefaction of organic matter and breakdown of toxic compounds (Fuhrmann et al., 2022; Chiam et al., 2021; Tan et al., 2021). The use of BSFFF boosts key soil health parameters, especially organic matter, nutrient concentrations, and key enzymes responsible for nutrient cycling such as dehydrogenase, β-glucosidase, urease, and phospho monoesterase in comparison with synthetic fertilizers and conventional compost (Esteves et al., 2022; Agustiyani et al., 2021), indicating the positive role of ICOF in boosting soil fertility, and restoring degraded soils.

Soil amendment with *Protaetia brevitarsis* frass significantly reduces the numbers of antibiotic resistance genes and mobile genetic elements in the rhizosphere and bulk soil, lettuce root and leaf endophytes compared to conventionally produced composts (Zhao et al., 2022), alleviating the spread risk of manure-borne antibiotic resistance genes to soil and plants. Soil amendment with BSFFF and exuviae boosts plant health through suppression of soil-borne pathogens such as *Fusarium spp., Ralstonia solanacearum, Rhizoctonia solani, Alternaria solani, B. cinerea, Sclerotinia sclerotiorum,* and *P. capsici* in cowpeas, beans and tomatoes (Kemboi et al., 2022; Quilliam et al., 2020; Choi and Hassanzadeh, 2019a, Choi and Hassanzadeh, 2019b). The antifungal activity of ICOF has been attributed to inhibition of mycelial growth by antifungal and/or anti-oomycetes compounds released after ICOF application (Arabzadeh et al., 2022). These attributes highlight the potential for use of ICOF as a source of novel biocontrol agents for plant pests and pathogens. Barragán-Fonseca et al. (2022) elucidated the perceived benefits of ICOF in the control soil-borne pathogens, and belowground and aboveground pests; but there is still very limited research evidence to this effect. Recent studies have revealed that some insects produce frass with volatiles that have the ability to attract pests or deter oviposition, such compounds could contribute to semiochemical-based management of pests using the attract-and-kill strategy (Revadi et al., 2021; Zhang et al., 2019).

## 8 Agronomic effectiveness of insect-composted organic fertilizers

Fertilizer is a critical input for food production, yet it has become more scarce and costly globally. For example, Africa consumes 8,267,000 t of inorganic fertilizer *per annum*, but most of it is currently imported (WFP, 2022). Fertilizer prices have increased by up to 113 % in East Africa, due to the Russia-Ukraine war that has disrupted global supply chain (WFP, 2022). A Kenya farmer must pay 58 % more for a bag of diammonium phosphate (DAP) compared to counterparts in Europe (FAO; IFAD; UNICEF; WFP and WHO, 2022). Consequently, ICOF is envisaged as an innovative local solution that could enhance the productivity of local food systems by addressing the challenges of scarce and costly fertilizer inputs as well as climate change.

Greenhouse and open-field studies have demonstrated tremendous increase in the yields of maize (7–27 %) (Beesigamukama et al., 2020a), tomatoes (22–135 %), kales (20–27 %) and French beans (38–50 %) (Anyega et al., 2021a, Anyega et al., 2021b), chili pepper and shallots (Quilliam et al., 2020), lettuce (36–54 %) (Dzepe et al., 2022) and ryegrass (17–218 %) (Menino et al., 2022) and *Brassica rapa* L. (1.1–26 folds) (Agustiyani et al., 2021) among others grown using BSFFF compared to commercial fertilizers and conventional composts. Soil amendment with 2–8 % BSF frass fertilizer has been found to increase rice yield by 44–196 % compared to unamended soil, and the rice yield increased with increase in rate of application (Wang et al., 2022).

*Brassica rapa* plants grown using composted BSF frass were 10 % larger than those grown using un-composted frass (Song et al., 2021), indicating the role of composting in increasing the quality of ICOF. Under the wonder multistorey gardening system, application of BSFFF has been found to increase the yields of kales (14–69 %) and Swiss chard (13–56 %) better than synthetic NPK fertilizer, and boost crop tolerance to water stress (Abiya et al., 2022). On the other hand, soil amendment with cricket frass improved leaf growth and height of *Amaranth tricolor* as good as synthetic NPK fertilizer, indicating that ICOF could a sustainable alternative to conventional fertilizers (Butnan and Duangpukdee, 2021).

Comparative studies have revealed the potential of frass fertilizers produced by crickets, desert locust and black soldier fly to increase the yield of bell pepper by 60 %, 47 % and 7 % compared to commercial NPK fertilizer. Furthermore, the yields of *Amaranthus dubius* grown using frass fertilizer from the African fruit beetle (*P. sinuata*) was 52–80 % and 4–19 % higher compared to those obtained using unfertilized soil and commercial organic fertilizer (SAFI), respectively.

Although, the benefits of ICOF on trees and perennial crops have not yet received adequate research attention, soil amendment with 4–6 % ICOF has been reported to increase growth, shoot dry matter, and shoot regrowth after cutting, and maintain 100 % survival of *Cichorium intybus* and *Plantago lanceolata* better than commercial organic fertilizer and biochar (Hodge and Conway, 2022). The same study also found that combined application of ICOF and biochar improved the effectiveness for pasture production, but ICOF amendment rates beyond 6 % were detrimental. Use of BSFFF has been reported to significantly boost the biomass yields of red clover and Italian ryegrass by up to 17 % compared to conventional composts and unamended soil (Fuhrmann et al., 2022), through improved soil fertility. On the other hand, soil amendment with insect frass fertilizer increased tree heights and root collar diameters of pines, birches, and oak grown on light sandy soils (Jung, 2008). Similar results were achieved using *Aqularia malaccensis* trees whereby application of ICOF increased the growth rate by 9–15 % compared to conventional organic fertilizers (Mohamad Amir Hamzah et al., 2021). Furthermore, application of frass fertilizers generated by superworms and mealworms improved the rooting and growth performance of *Hylocereus undatus*, but the performance of superworm frass was superior to that of mealworm frass (Gan et al., 2021).

Mealworm frass fertilizer has been found to enhance the growth, biomass accumulation, macronutrient uptake, and use efficiency of barley and ryegrass (Menino et al., 2022; Houben et al., 2020, Houben et al., 2021). Moreover, combined use of ICOF and synthetic fertilizers enhances nutrient use efficiency of different vegetable and cereal crops through a supplementary effect (Anyega et al., 2021a, Anyega et al., 2021b; Tanga et al., 2021a, Tanga et al., 2021b, Tanga et al., 2021c; Houben et al., 2020). Due to increased nutrient uptake and use efficiency, ICOF also enhances the nutritional quality of different crops through increased proteins, fiber and minerals (Agustiyani et al., 2021; Anyega et al., 2021a, Anyega et al., 2021b; Tanga et al., 2021a, Tanga et al., 2021b, Tanga et al., 2021c; Beesigamukama et al., 2020c). Studies have found that amendment of sandy and highly permeable soils with ICOF can increase the water use efficiency by 15–114 % compared to conventional compost, highlighting the critical role of ICOF in adapting food production to climate change shocks, and potential for extending crop production to less productive soils (Menino et al., 2022).

The other benefit of ICOF is related to boosting tolerance and resistance against biotic and abiotic stresses such as saline, water logged and waster stressed soils (Guo et al., 2021; Poveda et al., 2019). Use of ICOF enhances root growth and extension in crops such as *Beta vulgaris* var. *cicla* and *Brassica rapa*, through the supply of beneficial bacteria and growth hormones in the soil-plant system (Agustiyani et al., 2021; Poveda et al., 2019). Subsequently, the N fertilizer equivalence values of ICOFs such as BSFFF is higher than those of commercial organic fertilizers (1.3–38 folds) (Beesigamukama et al., 2020a), commonly used green manure plants, poultry litter, plant-based compost, and slurry from cattle and pigs (De Notaris et al., 2018; van Middelkoop and Holshof, 2017; Cavalli et al., 2016; Lalor et al., 2011; Bowden et al., 2007; Kimetu et al., 2004). This indicates the high suitability of ICOF as a complete or partial replacement for conventional organic or mineral fertilizers.

Frass fertilizer is also critical in bioremediation through alleviation of chemical and biological pollutants in the soil-plant system. Application of ICOF can considerably reduce the absorption (24–58 %) and transport (4–44 %) of heavy metals, such as cadmium and lead in rice plant system, thus improving food safety (Wang et al., 2022). On the other hand, the potential of insect chitin and chitosan in controlling bacterial diseases has been demonstrated with a suppression rate of up to 35 % (Kemboi et al., 2022). Kales and Swiss chard grown in soils amended with BSFFF had minimal infestation levels of aboveground pests such as aphids, diamondback moth, whiteflies and leaf miners compared to those grown using synthetic NPK fertilizer (Abiya et al., 2022). Ongoing studies spearheaded by the International Centre of Insect Physiology and Ecology (*icipe*) have demonstrated the high potential of chitin-fortified BSFFF to suppress soil-borne pests such as root knot nematodes, potato cyst nematodes, and onion root fly (*Delia radicum*) by up to 100 %, indicating the possibility of using chitin-fortified BSFFF as a bio-rational pesticide.

Economic assessments have revealed that production of crops using ICOF would yield up to 29–173 % higher net income compared to organic fertilizers (Beesigamukama et al., 2021a; Tanga et al., 2021a, Tanga et al., 2021b, Tanga et al., 2021c). Due to higher nutrient concentrations, lower application rates (1.3–2.5 t ha^−1^) or 10–15 % (volume/volume) of ICOF have been found sufficient for improved soil health and crop production compared to conventional organic fertilizers where higher application rates would be necessary (Agustiyani et al., 2021; Anyega et al., 2021a, Anyega et al., 2021b; Song et al., 2021; Beesigamukama et al., 2020c). It should be noted that excessive application of ICOF could inhibit plant growth due to nutrient toxicity (Chiam et al., 2021), thus use of optimum rates is key for improved yields. Due to the positive residual impacts on soil fertility, it may not be necessary to apply ICOF every growing cycle, because the residual soil nutrients can adequately support the production of subsequent crops (Chiam et al., 2021). This is crucial in reducing the high cost of fertilizers, which farmers normally incur seasonally while using conventional fertilizers.

It is crucial to note that the agronomic effectiveness of ICOF depends on the organic waste used in entomocomposting, presence or absence of frass treatment, and method of application. For example, the yield of lettuce grown using frass fertilizer derived from food wastes was 1.3–5.3-folds higher the value obtained using frass from okara, while application of frass fertilizer by side dressing was 1.6–6.8-folds more effective than soil drenching (Tan et al., 2021). On the other hand, use of untreated frass could hinder plant health by spreading pathogens to crops. Lettuce grown using untreated BSF frass was contaminated with pathogenic fungi and bacteria, indicating biological contamination and potential for disease transmission by immature and stable ICOF (Dzepe et al., 2022). It is, therefore, prudent that ICOF is properly treated before field application to reduce negative impacts on crops, soil, and environment, and improve the food safety. A composting period of five weeks was suggested based on physical and chemical indices of compost maturity (Beesigamukama et al., 2020b, Beesigamukama et al., 2021b), but it is not clear whether the same period is sufficient for eliminating pathogens that may be present in frass.

## 9 Policy and regulatory framework

Insect farming is at infant stages in most countries and requires standards to regulate mass rearing, commercialization, marketing and utilization of insect-based products. In East Africa, standards have been developed to regulate production, processing and marketing of edible insects and their products (RSB, 2021; Tanga et al., 2021c; KEBS, 2020; UNBS, 2019). However, these standards vary with region and continent, a critical bottleneck to global commercialization/marketing of insect-based products. For example, the European legislation allows only seven insect species, prohibits the use of animal wastes as feedstock and provides regulations on the use of frass a fertilizer (European Commission, 2018, European Commission, 2021; Tanga et al., 2021c). On the contrary, standards for African countries like Kenya, Uganda and Rwanda permit the consumption of all available edible insects in different communities, and use of all types of organic waste as feedstock but lack regulatory standards for frass fertilizer (RSB, 2021; KEBS, 2020; UNBS, 2019).

To abate the risks of contamination through the consumption of crops grown using ICOF, the European union regulation recommended a heat treatment of 70 °C for at least 1 h to minimize pathogen load and achieve the prescribed microbiological standards (European Commission, 2018). On the other hand, the standards of Kenya only advocate for proper treatment of insect waste products before disposal to prevent environmental contamination but do not provide threshold values and parameters to consider during waste sanitization (KEBS, 2020).

It is anticipated that the growing interest in the use of entomocompost will trigger formulation of regulatory standards for its production, quality assessment and commercialization in Africa, especially East Africa where regulatory standards for entomophagy already exist. This however, requires more evidence-based research and data on safety and benefits of ICOF to influence regulatory bodies and national governments and support scaling up.

## 10 Conclusion and research prospects

This review discusses the role of insect-based waste recycling technologies in a circular and green economy, and justifies opportunities that would contribute to better planetary health and boosting the sustainability of agricultural production, especially for smallholder farmers facing multiple production challenges in developing countries. The benefits of ICOF on boosting soil fertility, pathogen and disease suppression, crop yield and nutritional quality have been clearly demonstrated. Exploring the full potential of ICOF requires strategies for optimizing the bioconversion process to reduce nutrient losses, greenhouse gas emissions and contaminants to minimum levels, and establish standards and indices for assessing the quality of ICOF.

Increased uptake of ICOF will require capacity building and awareness creation among key stakeholders, including governments, agro-input dealers, farmers and private sector players in the fertilizer industry. Furthermore, diversification of ICOF products by developing powdered, granulated and liquid fertilizer products is critical to cater for different production requirements. Commercialization of ICOF will require legal frameworks to regulate the production, utilization and trade at a global scale. This can be achieved through multi-stakeholder collaborations.

Future research will be critical to determine the long-term benefits of ICOF on soil health, with emphasis on biennial and perennial crops to generate more accurate recommendations. Also, there is need to harness the biopesticide potential of ICOF for management of pests and diseases, induction of systemic plant resistance, and steering beneficial soil microbiota for improved plant defence and soil nutrient cycling.

Most studies on ICOF production and application have been conducted at lab/small scale, and the findings may not be easily transferred into largescale production systems. For example, Yakti et al. (2022) found that BSF larvae reared in small-scale boxes and at low larval densities had better nutritional quality but lower biomass weight, survival rate and longer development time than those from large-scale boxes. The same justification could be applied to agronomic experiments involving ICOF, most of which have been conducted under controlled environments (laboratory and/or greenhouse conditions). Thus, it is critical that promising research findings are demonstrated at large scale and/or field conditions to generate more accurate recommendation to steer technology uptake.

The following are the supplementary data related to this article.

Supplementary Table 1 Nutrient levels of BSF frass fertilizer generated from different organic wastes. Supplementary Table 1

## CRediT authorship contribution statement

**Dennis Beesigamukama:** Conceptualization, Data curation, Formal analysis, Investigation, Methodology, Resources, Software, Validation, Visualization, Writing – original draft, Writing – review & editing. **Chrysantus M. Tanga:** Conceptualization, Investigation, Methodology, Project administration, Resources, Software, Funding acquisition, Supervision, Validation, Visualization, Writing – original draft, Writing – review & editing. **Subramanian Sevgan:** Project administration, Supervision, Validation, Writing – review & editing. **Sunday Ekesi:** Project administration, Supervision, Validation, Writing – review & editing. **Segenet Kelemu:** Project administration, Supervision, Validation, Writing – review & editing.

## Declaration of competing interest

The authors declare that they have no known competing financial interests or personal relationships that could have appeared to influence the work reported in this paper.

## Data Availability

Data will be made available on request.
