# Supplementary material for: Waste to value: Global perspective on the impact of entomocomposting on environmental health, greenhouse gas mitigation and soil bioremediation
Source: Sci Total Environ. 2023 Dec 1;902:166067. doi: 10.1016/j.scitotenv.2023.166067 (PMC10594063; doi:10.1016/j.scitotenv.2023.166067)
Supplement: Supplementary Table 1 — Nutrient levels of BSF frass fertilizer generated from different organic wastes. [file mmc1.docx]

**Suppl. Table 1:** Nutrient levels of BSF frass fertilizer generated from different organic wastes

| Rearing substrate | N | P | K | Ca | Mg | Fe | Cu | Mn | Zn | References |
| --- | --- | --- | --- | --- | --- | --- | --- | --- | --- | --- |
|  | ………………..(%)………………………………. | | | | | (mg kg^-1^) | | | |  |
| Wheat bran, alfalfa, and cornmeal | 4.4 | 5.2 | 4.1 | 4.5 | 0.8 | 600 | 46.1 | ND | 410 | Setti et al. (2019) |
| Brewery spent grain and rice husks | 3.61 | 0.50 | 0.29 | 0.97 | 0.10 | 310 | 25 | 10 | 182 | Anyega et al. (2021); Tanga et al. (2021a) |
| Not specified | 3.3 | 3.4 | 2.4 | 0.4 | 1.0 | ND | ND | ND | ND | Gärttling et al. (2020) |
| Brewery spent grain | 2.1 | 1.16 | 0.17 | 0.16 | 0.19 | ND | ND | ND | ND | Beesigamukama et al. (2020a, 2020b) |
| Okara and wheat bran | 3.19 | 0.08 | 0.054 | 0.079 | 1.9 × 10-3 | 26.6 | 0.7 | 2.27 | 0.14 | Song et al. (2021) |
|  | 6.0 | 0.13 | 0.081 | 0.13 | 1.9 × 10-3 | 77.3 | 1.78 | 2.75 | 0.39 |  |
| Spent malted barley grain | 3.16 | 0.56 | 0.27 | 0.64 | 0.22 | 4100 | 12800 | 100 | 100 | Fuhrmann et al. (2022) |
| Okara (Soy bean pulp) | 5.15 | 0.19 | 0.029 | 1. 7 × 10-3 | 1.1 × 10-3 | 3.69 | 0.86 | 0.19 | 1.73 | Chiam et al. (2021) |

N = nitrogen, P = phosphorus, K = potassium, Ca = calcium, Mg = magnesium, Fe = iron, Cu = copper, Zn = zinc, S = sulphur, ND = not determined.
